# MicroRNA in Human Acute Kidney Injury: A Systematic Review Protocol

**DOI:** 10.1177/20543581211009999

**Published:** 2021-04-24

**Authors:** Adrianna Douvris, Dylan Burger, Rosendo A. Rodriguez, Edward G. Clark, Jose Viñas, Manoj M. Lalu, Risa Shorr, Kevin D. Burns

**Affiliations:** 1 Division of Nephrology, Department of Medicine and Kidney Research Centre, Ottawa Hospital Research Institute, The University of Ottawa and The Ottawa Hospital, ON, Canada; 2 Department of Cellular and Molecular Medicine, University of Ottawa, ON, Canada; 3 Department of Medicine, The University of Ottawa and The Ottawa Hospital, ON, Canada; 4 Department of Anesthesiology and Pain Medicine, Clinical Epidemiology and Regenerative Medicine Programs, Blueprint Translational Research Group, The Ottawa Hospital Research Institute, The University of Ottawa and The Ottawa Hospital, Canada

**Keywords:** acute kidney injury, systematic review, human, microRNA, biomarker

## Abstract

**Background:**

Acute kidney injury (AKI) is a common complication of hospitalization with high morbidity and mortality for which no effective treatments exist and for which current diagnostic tools have limitations for earlier identification. MicroRNAs (miRNAs) are small non-coding RNAs that have been implicated in the pathogenesis of AKI, and some miRNAs have shown promise as therapeutic tools in animal models of AKI. However, less is known about the role of miRNAs in human AKI.

**Objective:**

To evaluate the role of miRNAs in human subjects with AKI.

**Design:**

Systematic review and meta-analysis

**Measurements:**

Quantification of miRNA levels from human blood, urine, or kidney biopsy samples, and measures of renal function as defined in the study protocol.

**Methods:**

A comprehensive search strategy for Ovid MEDLINE All, Embase, Web of Science, and CENTRAL will be developed to identify investigational studies that evaluated the relationship between miRNA levels and human AKI. Primary outcomes will include measurements of kidney function and miRNA levels. Study screening, review and data extraction will be performed independently by 2 reviewers. Study quality and certainty of evidence will be assessed with validated tools. A narrative synthesis will be included and the possibility for meta-analysis will be assessed according to characteristics of clinical and statistical heterogeneity between studies.

**Limitations:**

These include (1) lack of randomized trials of miRNAs for the prevention or treatment of human AKI, (2) quality of included studies, and (3) sources of clinical and statistical heterogeneity that may affect strength and reproducibility of results.

**Conclusion:**

Previous studies of miRNAs in different animal models of AKI have generated strong interest on their use for the prevention and treatment of human AKI. This systematic review will characterize the most promising miRNAs for human research and will identify methodological constraints from miRNA research in human AKI to help inform the design of future studies.

**Systematic review registration:**

PROSPERO CRD42020201253

## Abrégé

**Contexte:**

L’insuffisance rénale aiguë (IRA) est une complication fréquente des hospitalisations avec morbidité et mortalité élevées. Il n’existe aucun traitement efficace contre l’IRA et les outils diagnostiques actuels qui permettent son dépistage précoce comportent des limites. Les microARN (miARN) sont de petits ARN non codants ayant été impliqués dans la pathogenèse de l’IRA; certains d’entre eux se sont révélés prometteurs comme outils thérapeutiques dans les modèles animaux de l’IRA. Le rôle des miARN dans l’IRA chez l’humain est cependant moins connu.

**Objectif:**

Évaluer le rôle des miARN chez les sujets humains atteints d’IRA.

**Type d’étude:**

Examen systématique et méta-analyze

**Mesures:**

La quantification des taux de miARN chez l’humain à partir d’échantillons de sang, d’urine ou de biopsie rénale, et mesure de la fonction rénale telle que définie dans le protocole de l’étude.

**Méthodologie:**

Une stratégie de recherche exhaustive des bases de données Ovid MEDLINE All, Embase, Web of Science et CENTRAL sera élaborée afin de répertorier les études expérimentales ayant évalué la relation entre les taux de miARN et l’IRA chez l’humain. Les principaux critères d’évaluation comprendront la mesure de la fonction rénale et des taux de miARN. Deux examinateurs procéderont de façon indépendante à la sélection des études, à leur examen et à l’extraction des données. La qualité des études et la robustesse des données seront évaluées à l’aide d’outils validés. Une synthèse descriptive sera incluse et la possibilité d’une méta-analyze sera évaluée en fonction des caractéristiques de l’hétérogénéité clinique et statistique entre les études.

**Limites:**

Les limites de l’étude concernent notamment (i) le manque d’essais randomisés examinant les miARN pour la prévention ou le traitement de l’IRA humaine; (ii) la qualité des études incluses; et (iii) les sources d’hétérogénéité clinique et statistique susceptibles d’affecter la robustesse et la reproductibilité des résultats.

**Conclusion:**

Des études antérieures sur les miARN dans différents modèles animaux de l’IRA ont suscité un vif intérêt pour leur utilization dans la prévention et le traitement de l’IRA chez l’humain. Cet examen systématique caractérisera les miARN les plus prometteurs pour la recherche sur l’IRA humaine et définira les contraintes méthodologiques de telles études, ce qui aidera à orienter la conception des études futures.

## Background

Acute kidney injury (AKI) refers to a rapid decline in kidney function. Acute kidney injury affects up to 20% of hospitalized patients particularly in those admitted to intensive care units (ICU).^1^ Patients who develop AKI have an increased risk of death^1^ and are more likely to have long term adverse outcomes if they recovered from AKI. In a meta-analysis of 82 studies with 2 million hospitalized adults followed for minimum 1 year, AKI was associated with a nearly 2-fold increased risk of death, a 3-fold increased risk of new or progressive chronic kidney disease (CKD), and a 4-fold increased risk of kidney failure compared to patients without AKI.^2^ The incremental cost of AKI in Canada is estimated to exceed CAN$200 million per year, and greater severity AKI is associated with incremental increases in both hospital length of stay and costs.^3^ Despite the health and economic burden of AKI, no effective interventions currently exist for treatment,^4^ preventative measures are limited,^5^ and current diagnostic tools including serum creatinine and urine output have major limitations for earlier identification.^6^

AKI can be caused by different mechanisms, including ischemia-reperfusion injury (IRI), sepsis, and nephrotoxins. At the cellular level, proximal tubular and endothelial cell injury involve pathways in inflammation, apoptosis, angiogenesis, and fibrosis.^7^ MicroRNAs (miRNAs) are small non-coding RNAs, well conserved across species, that regulate gene expression at the post-transcriptional level by binding the 3′-untranslated region (UTR) of messenger RNA (mRNA), thereby inhibiting mRNA translation and promoting mRNA degradation.^8^ Thus far, more than 1500 miRNAs have been identified in humans and have been found to be involved in many biological processes including cell cycle regulation, apoptosis, hypoxia, metabolism, immunity, and oncogenesis.^7^ Over the last 10 years, there has been an expanding effort to evaluate the role of miRNAs in AKI. This is reflected by the increasing annual number of publications on this topic over this time period in PubMed: from 4 publications in 2010, to an average of 50-60 publications annually over the last 5 years.

Several miRNAs have been studied in animal models of AKI—the most frequent of which is miR-21—for their expression patterns, mechanisms of action and therapeutic potential.^9^ For instance, Song *et al* evaluated the effect of miR-21 knockdown on AKI using in vitro hypoxia/re-oxygenation and in vivo mouse IRI models.^10^ These knockdown studies suggest that miR-21 protects against ischemic AKI. miR-21 knockdown intensified ischemia-induced renal epithelial cell injury by (1) enhanced apoptosis through the phosphatase and tensin homolog (PTEN)/Akt/mammalian target of rapamycin (mTOR)/hypoxia-inducible factor (HIF) pathways and (2) increased inflammation by promoting dendritic cell maturation.^10^

Pre-clinical research is an important step for the translation of basic science to human studies, and a systematic review of miRNAs as therapy for pre-clinical models of AKI is underway.^11^ Although miRNAs are showing promise as therapies in pre-clinical AKI models, much less is known about their potential as a therapy to prevent or treat AKI in humans.^12^ Furthermore, pre-clinical studies have not readily translated to human application and study design flaws are a contributing factor. For example, many studies of IRI-AKI are conducted in male animals.^13^ However, animal experimental models have shown that female sex confers protection against IRI-AKI,^14-17^ an observation that has also been noted from observational studies and meta-analysis in humans.^18,19^

Currently, no clinical trials on the therapeutic use of miRNA in human AKI have been registered (clinicaltrials.gov, https://www.isrctn.com/), but several miRNAs have been evaluated as biomarkers of AKI and other kidney diseases.^12^ In cardiac surgery and critically ill patients, small studies that measured urinary or plasma levels of miRNAs as a prognostic marker of AKI have reported conflictive results and this highlights the complexity of miRNA pathways.^7^ One such example is miR-21, whose levels have been shown to be increased in the urine and plasma of critically ill and cardiac surgery patients with AKI,^20-22^ yet another study of cardiac surgery patients found that lower pre-operative plasma miR-21 levels conferred an increased risk of developing cardiac surgery-associated AKI.^23^ This systematic review will evaluate many aspects of miRNAs related to human AKI, including an improved understanding of the pathophysiology related to different AKI causes, and will identify methodological limitations, sources of heterogeneity and outcome measure differences, which will help inform the planning and design of clinical trials.

## Methods

### Study Design

This systematic review will be reported in accordance with Preferred Reporting Items for Systematic Reviews and Meta-Analyses (PRISMA) statements, and the Cochrane Handbook for Systematic Reviews of Interventions will be used as best-practice guidance.^24,25^ The study protocol was written in accordance with PRISMA for protocols (PRISMA-P) and is registered with the International Prospective Register of Systematic Reviews (PROSPERO CRD42020201253). A populated PRISMA-P checklist is provided in supplementary file 1.

### Objectives

The primary aim is to evaluate the role of miRNAs in human AKI. The secondary aims will be to evaluate the association between miRNAs and renal outcomes including AKI severity, the incidence of dialysis-requiring AKI, renal recovery, and in-hospital mortality. The relationship between timing of miRNA measures in AKI and clinical outcomes, and the effect of sex on miRNA expression in AKI will be evaluated if possible.

### Types of Studies

Observational and interventional studies (randomized and non-randomized) evaluating miRNAs in human AKI that meet the inclusion criteria below will be included. Narrative reviews, editorials, and case reports will be excluded.

#### Population

Studies of human subjects (adult and pediatric populations) with both dialysis-requiring and non-dialysis-requiring AKI will be included. AKI will be defined broadly using RIFLE and AKIN definitions outlined in the KDIGO clinical practice guidelines for AKI.^5^ All AKI causes will be included (ie, etiology), but if there are sufficient number of studies, subgroup analyses will be conducted to determine any differences in the etiology of AKI. Studies of patients with end-stage kidney disease treated with kidney replacement therapy (maintenance dialysis) will be excluded.

Studies involving non-human animal and cell culture models will be excluded.

#### Exposure

The exposure of interest is AKI. Studies will characterize the relationship between the presence of AKI, AKI severity and etiology, and miRNA levels.

#### Comparator(s)

Studies will be comparing miRNA levels in patients with AKI to those without AKI. In the event there is no comparator group, studies may compare miRNA levels with differing severity of AKI.

#### Confounders

These include any variables that can influence miRNA levels. As miRNAs affect a range of biological processes and their expression is dysregulated in many diseases, confounders include AKI cause and CKD,^12^ diabetes mellitus,^26^ infection/sepsis,^27^ cancer,^28,29^ and medications (ie anticoagulants^30^ and anti-cancer agents).^29^ Age will also be explored as a confounding factor.

#### Outcomes

(a) *Primary outcome*: miRNA levels from blood, urine, or kidney tissue in association with AKI. miRNA quantification methods may include but are not limited to quantitative PCR, microarray, or quantification using fluorescence *in situ* hybridization.
(b) *Secondary outcomes*: (1) Measurement of renal function by serum Cr, BUN or urea, or urine volume; (2) biomarkers of AKI^31^ (ie neutrophil gelatinase-associated lipocalin (NGAL), kidney injury molecule 1 (KIM-1) or others), and kidney structural changes by kidney biopsy for histological analyses if available; (3) measures of association between miRNA levels and other outcomes of interest: (i) severity of AKI, (ii) dialysis-requiring AKI, and (iii) complete or partial renal recovery (broad criteria due to lack of uniform definition^32^ includes serum Cr < 1.5× baseline, or, no longer requiring renal replacement therapy, or, serum Cr decreases but does not return to <1.5× baseline), and (iv) in-hospital mortality.
(c) *Exploratory outcomes*: (i) the association between sex and miRNA levels in AKI, (ii) between miRNA level and AKI cause, and (iii) timing of miRNA measurement and clinical outcomes.

### Search Strategy

We performed an initial search using PubMed for prior systematic reviews on this topic. We also searched PROSPERO to identify currently registered systematic reviews. We did not identify any prior or ongoing systematic review.

We will implement a comprehensive search strategy in collaboration with a research librarian (provided in supplementary file 2). The search will include terms related to acute kidney injury, miRNA, and extracellular vesicles (which may contain miRNA as cargo).

We will search the following databases from years 1947 to present:

- Ovid MEDLINE All
- Embase
- CENTRAL (Cochrane Central Register of Controlled Trials)
- Web of Science

The search will be supplemented by manually scanning the reference list of all included studies for additional material. Editorials, review articles, case reports, patent applications, and studies involving only animal or in-vitro experiments will be excluded. Abstracts from the last 5 years from the American Society of Nephrology scientific meetings will be reviewed. A search of clinicaltrials.gov for any contemporary or previous protocols will be conducted to verify eligible studies.

### Study Selection

Titles and abstracts initially identified from our search will be exported to Covidence for independent screening by at least 2 investigators. Duplicate citations will be removed.

The following requirements must be met for an article to be considered for full text review:

- Original study (observational or interventional) of human AKI with miRNA expression measures. We will exclude non-original research (ie review or editorials), and pre-clinical studies.
- Possible causes of AKI include but are not limited to (1) pre-renal, hepatorenal, or cardio-renal syndrome; (2) acute tubular necrosis; (3) major surgery (ie cardiac, vascular, intra-abdominal); (4) shock; (5) sepsis; (6) nephrotoxins; (7) acute obstruction; (8) acute interstitial nephritis; (9) acute glomerulonephritis; (10) malignancy-associated AKI and (11) AKI in renal transplant recipients including delayed graft function and acute rejection. Studies of patients with end-stage kidney disease treated with kidney replacement therapy (maintenance dialysis) will be excluded.
- Studies in English and French languages will be included.
- Studies that do not specifically address miRNA or kidney function will be excluded.

The 2 reviewers will also document the primary reason for article exclusion after full text review. Any discrepancies between included and excluded studies between the 2 independent reviewers will be discussed for a consensus decision. If a consensus cannot be reached, a third reviewer will provide an independent opinion. We will calculate the kappa statistic for inter-reviewer reliability.

### Data Extraction

A data extraction form will be created and piloted prior to duplicate data extraction by 2 independent reviewers. The data extracted will include the following:

- *Study characteristics*: authors, journal information, publication year, geographic location, in-patient setting (ward, ICU, post-cardiac surgery, transplant), study design, type of publication (abstract or full manuscript).
- *Population characteristics*: Age, sex, ethnicity, comorbidities, AKI setting (ie ICU, post-cardiac surgery, etc), admission diagnosis, AKI cause.
- Type of exposure and comparator:

○ Type of kidney insult responsible for AKI if applicable to the study (ie cardiac surgery, sepsis, nephrotoxin) and timing of AKI if available.
○ Control group characteristics
○ Duration of follow-up.

- Outcomes:

○ *Primary outcome*: miRNA characteristics: variant, type of sample (blood, urine, kidney tissue), timing of measurement relative to AKI, method of miRNA measurement including assay and instrument. Normalization methods and measurement units for miRNA expression will be identified.
○ *Secondary and exploratory outcomes*: (i) measures of renal function and markers of AKI as described above; (ii) AKI severity, incidence of dialysis-requiring AKI, in-hospital mortality, renal recovery; (iii) comparison of miRNA levels with presence of AKI, renal outcomes, and sex; (iv) relationship between miRNA and AKI cause; and (v) timing of miRNA measurement and clinical outcomes

If information is missing from a study, attempts will be made to contact the study authors. The data will be exported from Covidence for data analysis.

### Risk of Bias and Quality Assessment

Two independent reviewers will assess the included studies for potential bias and quality. Non-randomized studies will be evaluated for risk of bias using the Newcastle-Ottawa Scale (NOS),^33^ which evaluates study group selection, comparability, and outcomes. If randomized studies meeting inclusion criteria are identified, risk of bias will evaluated with the Cochrane Handbook Risk of Bias Assessment Tool.^34^ Any disagreements will be resolved by discussion, and, if not possible, then a third reviewer.

### Data Synthesis and Analysis

The decision to perform a meta-analysis on the primary outcome will depend on the assessment of statistical and clinical heterogeneity. Assessment of clinical heterogeneity between studies will be based on miRNA variant, sample type (blood, urine, kidney biopsy), miRNA normalization methods, AKI cause, and timing of measurement relative to AKI if available. We will assess statistical heterogeneity using the *I*^2^ statistic.^35^ If statistical heterogeneity between studies is high (*I*^2^ > 50% and deemed to represent considerable heterogeneity), then data will be reported descriptively for the outcomes of interest. In these cases, we will provide a narrative synthesis of included studies using the Synthesis Without Meta-analysis (SWiM) reporting guideline as a framework.^36^ If clinical and statistical heterogeneity are acceptable, studies with similar outcomes measures and miRNAs will be pooled to calculate pooled weight effect estimates using the inverse variance method, and data will be modeled according to the DerSimonian-Laird Method (random effects model).^37^

If sufficient studies are eligible, we will report on likely confounders of miRNA expression, including cause of AKI, underlying comorbidities (ie CKD, diabetes mellitus, cancer), medications, and acute medical conditions (ie type of surgery, infection, shock, acute coronary syndrome). We will assess the quality of control for confounders by reporting whether studies: (1) had balanced groups, (2) matched subjects for the confounders, and (3) adjusted for the confounders in their statistical analyses. Where possible, we will perform sub-group analyses listed in the following.

### Subgroup Analysis

If data are available from included studies, subgroup analyses will include the following:

(a) Population characteristics:
a. Pediatric vs adult population
b. Male vs female sex
(b) Exposures:
a. AKI cause
b. Severity of AKI
(c) Outcomes:
a. Timing of miRNA measurement relative to AKI: categorized as (1) prior to AKI onset, (2) early AKI (within 48 hours of injury), or (3) late (after 48 hours)
b. Sample type (blood, urine, or tissue)
c. Requirement for renal replacement therapy (yes/no)
d. Renal recovery (yes/no)
e. In-hospital mortality (yes/no)

Assessment of reporting biases will be performed on the primary outcome by constructing a funnel plot if an adequate number of studies is identified.^35^

Data analysis will be performed using RevMan 5.3.

### Assessing the Quality of Evidence

The quality of evidence for the primary outcome will be assessed as “very low” to “high” in accordance to the Grading of Recommendations Assessment, Development and Evaluation (GRADE) Workgroup.^38^ Limitations of included studies will also be identified with suggestions for improvement where possible.

## Discussion

AKI occurs frequently in hospitalized patients and is associated with increased morbidity and mortality. There is an important need for tools for early detection of AKI, and the development of novel therapies for its prevention and treatment. Pre-clinical miRNA studies in AKI have shown promise as potential AKI markers and therapies. Those studies have uncovered plausible biological mechanisms implicating several miRNAs in the pathogenesis of AKI. More than 50 different miRNAs are differentially expressed in AKI,^8^ but their significance in human AKI is unclear. In addition, translating pre-clinical AKI research to clinical practice has proven to be challenging due to pre-clinical methodological issues that are beyond the scope of this review, and important differences between experimental AKI models and human AKI.^13^ For instance, rodents and other mammals lack modeling for common medical comorbidities such as CKD, diabetes mellitus, or hypertension which impact AKI prognosis. There are sex-specific differences in AKI susceptibility, thus most pre-clinical studies are conducted in male animals. Of note, gender may have effects independently of biological sex;^39^ however, this is unlikely to be reported. Pre-clinical models are also not truly representative of AKI in humans which is often multifactorial in the setting of systemic illness. Another consideration is the timing of intervention in human AKI: with the exception of specific circumstances (ie planned cardiac surgery), the onset of AKI is often unknown or identified late.

A systematic review of miRNAs in human AKI will identify the miRNAs selected for study in humans thus far, the reason for their selection (ie biological mechanism, pre-clinical results) and may uncover other miRNAs that have not yet been studied in pre-clinical models, creating new research opportunities for scientists. Our systematic review of miRNAs in experimental AKI models^11^ will serve to facilitate the comparison of miRNAs in pre-clinical versus human AKI. Further, a systematic review of miRNAs in human AKI will address the heterogeneity and controversy among studies of miRNA in AKI by identifying the directionality and magnitude of miRNA associations with AKI. This information can be used to determine if pre-clinical studies support the direction of effects seen in human AKI, and, if quantitative analysis of effect size is possible, can be helpful to calculate an estimated sample size in future clinical trials. It will also identify important limitations from existing studies that could be used by researchers to improve future clinical trial design. These include methodological limitations, sources of heterogeneity, and confounders that could impact accuracy of results, and outcome measures. Overall, this information will inform researchers and clinicians on better planning further well-designed studies for both AKI diagnosis and treatment.

There are limitations to this planned systematic review. We do not expect to identify human clinical trials of miRNA delivery systems for the prevention or treatment of AKI. Our data will come from descriptive clinical studies which will not provide mechanistic information on their own without pre-clinical experimental models. The timing of miRNA measures relative to AKI may not be possible to control in some clinical settings which may affect the primary outcome, but timing will be considered in the analysis. Finally, small sample sizes and study design with respect to case and control cohorts may also affect the strength and reproducibility of results.

The field of miRNA therapeutics is beginning to evolve from pre-clinical studies to phase I/II clinical trials in other disciplines. In Oncology, the first such phase I trial used a miR-34 mimic (MRX34) with an intravenous nanoparticle liposomal delivery system for 85 adult patients with refractory advanced solid tumors.^40^ Although the study was able to show miR-34 delivery to tumors and dose-dependent target gene modulation, it was stopped early due to serious immune-related adverse effects.^41^ In the treatment of hepatitis C infection, a retrospective follow up analysis of a phase 2a multicentre trial found that the locked nucleic acid antisense inhibitor of miR-122 miravirsen had a sustained virological response in 58% (7/12) of trial participants and no significant adverse effects over a 35-month follow-up period.^42^ Given miRNA therapeutics are being studied in clinical trial settings for other illnesses, this could be a promising direction for the prevention and treatment of AKI.

## Conclusions

A systematic review and meta-analysis of the role of miRNAs in human AKI will be conducted. This study will identify and characterize the most promising miRNAs for further AKI research and methodological limitations from current miRNA research in human AKI that will help improve the design of future clinical and experimental studies in this area.

## Supplemental Material

sj-docx-1-cjk-10.1177_20543581211009999 – Supplemental material for MicroRNA in Human Acute Kidney Injury: A Systematic Review Protocol Click here for additional data file. Supplemental material, sj-docx-1-cjk-10.1177_20543581211009999 for MicroRNA in Human Acute Kidney Injury: A Systematic Review Protocol by Adrianna Douvris, Dylan Burger, Rosendo A. Rodriguez, Edward G. Clark, Jose Viñas, Manoj M. Lalu, Risa Shorr and Kevin D. Burns in Canadian Journal of Kidney Health and Disease

sj-docx-2-cjk-10.1177_20543581211009999 – Supplemental material for MicroRNA in Human Acute Kidney Injury: A Systematic Review Protocol Click here for additional data file. Supplemental material, sj-docx-2-cjk-10.1177_20543581211009999 for MicroRNA in Human Acute Kidney Injury: A Systematic Review Protocol by Adrianna Douvris, Dylan Burger, Rosendo A. Rodriguez, Edward G. Clark, Jose Viñas, Manoj M. Lalu, Risa Shorr and Kevin D. Burns in Canadian Journal of Kidney Health and Disease
