# Supplementary material for: MicroRNA in Human Acute Kidney Injury: A Systematic Review Protocol
Source: Can J Kidney Health Dis. 2021 Apr 24;8:20543581211009999. doi: 10.1177/20543581211009999 (PMC8072838; doi:10.1177/20543581211009999)
Supplement: sj-docx-2-cjk-10.1177_20543581211009999 – Supplemental material for MicroRNA in Human Acute Kidney Injury: A Systematic Review Protocol [file sj-docx-2-cjk-10.1177_20543581211009999.docx]

**Supplementary file: Samples Ovid MEDLINE All Search Strategy**

1 exp MicroRNAs/ (237535)

2 (MicroRNA* or Micro RNA* or non coding rna* or noncoding rna* or Small Temporal rna* or miRNA* or mir).tw,kw. (323176)

3 mir.kf. (20042)

4 (microparticle* or ectosome* or exosome* or microvesicle* or extracellular vesicle*).tw,kw. (90546)

5 cell-derived microparticles/ or exosomes/ (39283)

6 or/1-5 (418855)

7 exp Acute Kidney Injury/ (136854)

8 (acute adj2 (renal or kidney)).tw. (137953)

9 ((acute or ischem* or ischaem* or reperfusion) and (kidney or renal)).kf. (11332)

10 ((ischem* or ischaem* or reperfusion) adj3 (renal or kidney)).tw. (24330)

11 aki.tw,kw. (36604)

12 ((acute or toxic or contrast induced) adj2 nephropath*).tw. (7948)

13 ((acute or toxic or contrast induced) and nephropath*).kf. (707)

14 nephrotoxi*.tw,kw. (61088)

15 tubular necrosis.tw,kw. (12348)

16 Delayed Graft Function/ (7804)

17 delay* graft function*.tw,kw. (10923)

18 or/7-17 (277406)

19 6 and 18 (1884)

20 exp animals/ not humans/ (17464765)

21 19 not 20 (1004)

22 limit 21 to (english or french) (977)

**23 22 use medall (555)**

24 exp microRNA/ (237535)

25 (MicroRNA* or Micro RNA* or non coding rna* or Small Temporal rna* or miRNA* or mir).tw. (300243)

26 exosome/ (27367)

27 membrane microparticle/ (5241)

28 (microparticle* or ectosome* or exosome* or microvesicle* or extracellular vesicle*).tw. (87850)

29 24 or 25 or 26 or 27 or 28 (396393)

30 exp Acute Kidney Injury/ (136854)

31 (acute adj2 (renal or kidney)).tw. (137953)

32 aki.tw. (36126)

33 kidney ischemia/ (10844)

34 contrast induced nephropathy/ (4855)

35 ((acute or toxic or contrast induced) adj2 nephropath*).tw. (7948)

36 ((ischem* or ischaem* or reperfusion) adj3 (renal or kidney)).tw. (24330)

37 nephrotoxicity/ (64304)

38 kidney tubule necrosis/ (4585)

39 tubular necrosis.tw. (12086)

40 delayed graft function/ (7804)

41 delay* graft function.tw. (10690)

42 or/30-41 (287746)

43 29 and 42 (1775)

44 (exp animal/ or exp invertebrate/ or nonhuman/ or animal experiment/ or animal tissue/ or animal model/ or exp plant/ or exp fungus/ or preclinical study/) not (exp human/ or human tissue/) (13129640)

45 43 not 44 (1323)

46 limit 45 to (english or french) (1293)

**47 46 use emczd (841)**

48 exp MicroRNAs/ (237535)

49 (MicroRNA* or Micro RNA* or non coding rna* or noncoding rna* or Small Temporal rna* or miRNA* or mir).tw,kw. (323176)

50 mir.kw. (26692)

51 (microparticle* or ectosome* or exosome* or microvesicle* or extracellular vesicle*).tw,kw. (90546)

52 cell-derived microparticles/ or exosomes/ (39283)

53 or/48-52 (418770)

54 exp Acute Kidney Injury/ (136854)

55 (acute adj2 (renal or kidney)).tw. (137953)

56 ((acute or ischem* or ischaem* or reperfusion) and (kidney or renal)).kw. (27555)

57 ((ischem* or ischaem* or reperfusion) adj3 (renal or kidney)).tw. (24330)

58 aki.tw,kw. (36604)

59 ((acute or toxic or contrast induced) adj2 nephropath*).tw. (7948)

60 ((acute or toxic or contrast induced) and nephropath*).kw. (2083)

61 nephrotoxi*.tw,kw. (61088)

62 tubular necrosis.tw,kw. (12348)

63 Delayed Graft Function/ (7804)

64 delay* graft function*.tw,kw. (10923)

65 or/54-64 (282405)

66 53 and 65 (1895)

**67 66 use cctr (31)**

68 22 or 47 or 67 (1477)

69 remove duplicates from 68 (1066)

70 69 use medall (548)

71 69 use emczd (497)

72 69 use cctr (21)
